# Systems biology and biological systems diversity for the engineering of microbial cell factories

**DOI:** 10.1186/1475-2859-6-35

**Published:** 2007-11-20

**Authors:** Pau Ferrer

**Affiliations:** 1 Department of Chemical Engineering, Universitat Autònoma de Barcelona, 08193-Bellaterra (Cerdanyola del Vallès), Spain

Metabolic engineering was originally conceived as a systems approach to optimise biotechnologically desired traits of microbes and higher cells [1]. *Microbial Cell Factories *has published several review and research articles on this field over the past recent years [2-8]. Although clear breakthroughs have been achieved in the past, progress in metabolic engineering has been largely limited to individual pathways or relatively simple networks. Engineering of complex metabolic networks has been hampered by the insufficient biological information and global analytical tools.

Systems biology is increasingly generating a quantitative knowledge base of cell physiology, offering, for the first time, insights into molecular/cellular processes and function at a cell-wide scale. In order for metabolic/cellular engineers to embrace the potential that systems biology offers, an understanding of a variety of analytical and mathematical/computational tools is required [9-12]. However, whilst our knowledge on the systems components (genes, proteins, metabolites) has increased significantly, data integration in computer models with appropriate mechanistic and molecular detail to enable *in silico *experiments of sufficient predictive capability is still limiting the increase in the success rate of microbial cell factories engineering strategies.

*Microbial Cell Factories *is already contributing to individual systems biology-driven technological and methodological advances, experimental (at the transcriptomic [13-15], proteomic [16] and metabolomic/fluoxomic levels [17-20]) or computational [21,22]. Whilst strengthening this trend, we would also like to expand our field of interest to the integration of experimental data with computational and theoretical methods by encouraging the publication of research and review articles covering core aspects of the application of systems biology to the engineering of microbial cell factories (i.e. metabolic or cellular engineering). Moreover, the growing number of host cell systems being explored as factories, the continuous improvement of genetic tools, progress in *de novo *synthesis of increasingly complex biological entities (synthetic biology [23]) and, the growing diversity of bioproducts, is creating an emerging interest to extend our knowledge base to cell factories other than the classic model organisms. Also, a comparative analysis amongst different organisms is expected to yield new insights in cellular processes and function.

While of general interest in the green and white biotechnology, we believe our initiative will also contribute to fulfil the changing needs within highly specialized technical and scientific areas in biomedicine and biotechnology.
